# An economic evaluation of a mobile text messaging intervention to improve mental health care in resource-poor communities in China: a cost-effectiveness study

**DOI:** 10.1186/s12913-020-05855-6

**Published:** 2020-10-28

**Authors:** Rubee Dev, Jinghua Li, Donglan Zhang, Yiyuan Cai, Chun Hao, Fengsu Hou, Ruixin Wang, Meijuan Lin, Dong (Roman) Xu

**Affiliations:** 1 Sun Yat-sen University Global Health Institute (SGHI), School of Public Health & Institute of State Governance, Sun Yat-sen University, Guangzhou, China; 2 Department of Health Policy and Management, College of Public Health, University of Georgia, Athens, GA USA; 3 Department of Preventive Medicine and Maternity and Child Care, School of Public Health, Guizhou Medical University, Guiyang, China; 4 Department of Public Mental Health, Shenzhen Kangning Hospital, Shenzhen, Guangdong China; 5 ACACIA Lab and Department of Health Management, School of Health Management, Southern Medical University, Guangzhou, China

**Keywords:** Cost-effectiveness, Cost-utility, Mental disorder, Schizophrenia

## Abstract

**Background:**

Severe mental disorders, a leading cause of disability has become a major public health problem. In order to promote mental health, a series of programs have been promulgated by the Chinese government. However, economic evaluations of such programs are lacking. The purpose of this study is to develop and validate an economic model to assess the cost and health outcomes of the LEAN (Lay health supporters, E-platform, Award, and iNtegration) program, and to perform an economic evaluation of LEAN versus the nationwide community-based mental health program that provides free antipsychotic medications.

**Methods:**

A cost-effectiveness and cost-utility analysis of the LEAN intervention will be performed. A Markov model will be developed, validated and used to assess and compare the costs and outcomes for the LEAN intervention versus nationwide community-based mental health program. The calculated sample size is 258 participants for the analysis. A societal perspective will be applied with the time horizon of 1-year after the termination of the LEAN program. The cost-utility will be measured primarily using Quality Adjusted Life Years and the cost-effectiveness will be measured using number of relapses and number of re-hospitalizations avoided 6-month after the intervention. Univariate and probabilistic sensitivity analysis will be conducted for the analysis of uncertainty.

**Discussion:**

If proven cost-effective, this study will contribute to the nationwide implementation of the program, not only for schizophrenia but for all kind of severe mental disorders. Markov model developed as part of the study will benefit potential researchers in analyzing cost-effectiveness of other programs. The Chinese context of the study may limit the generalizability of the study results to some extent.

**Trial registration:**

This study was registered in a Chinese Clinical Trial Registry (ChiCTR2000034962) on 25 July 2020.

## Background

With a rapid change in the socio-economic development over the past three decades, the rise in the incidence rates of severe mental disorders has become a major public health problem in China [1]. Severe mental disorders, which include schizophrenia, bipolar disorder and severe depression are complex psychiatric disorders that manifest into disabling conditions of hallucinations, delusions, and disordered thinking and behavior [2]. Due to their disabling effects, it has devastating costs to patient, families, and society. In China, the burden of mental disorders account for 13% of all non-communicable diseases burden and is expected to increase by 10% by the year 2025 [3], warranting an urgent prioritization of programs focused on targeted prevention and effective treatment.

Effective treatment is available for mental disorders, but the shortage of mental health professionals and nonadherence to treatment have led to large treatment gaps in China [4, 5]. A systematic review of the studies that had used the internet- and mobile-based interventions to overcome the barrier in providing mental healthcare showed clinically and cost-effective results [6]; however, no studies included in the review were conducted among the Chinese population. To understand the potential role of mobile intervention to help address the challenge in providing mental healthcare, our study team conducted a randomized controlled trial to leverage the power of family members by using mobile texting to assist in formal patient care in a resource constraint rural area in China. Our approach, called “LEAN” (Lay health supporters, E-platform, Award, and iNtegration of services with the health system), involved the recruitment of lay health supporters aided by text messages for medication reminders, health education, monitoring of early signs of relapses, and facilitating linkage to primary healthcare to the patients with schizophrenia [7, 8]. LEAN intervention adapted the Health Belief Model (HBM) to target individual components of LEAN from the empirical literature on task shifting and mHealth to improve patient adherence to medication [9]. With the increasing prevalence of the severe mental disorder, there is also an increase in the burden experienced by caregivers and health care providers. Nonadherence to antipsychotic medication is one of the major concerns among these patients that increases the risk of relapse and re-hospitalization [10], suggesting a need of the development of strategies that would reduce medication nonadherence [5, 11].

A nationwide mental health program (the 686 program) was launched in China in 2005 [12] with an aim to address the challenges around the lack of integration between hospital and community health care for managing severe mental disorders. The program moved specialty mental health services into community settings providing outreach services into the community. Although the program has been able to improve access to care, it has not been able to reach its ambitious goals due to enormous challenges in the delivery of the program, greater commitment of resources, and limited available services in the community [12, 13]. Potentially, a simple, easy-to-implement and sustainable service model that can be efficient even in the presence of few mental health professionals and small mental health budgets like LEAN intervention can bring significant benefits to China’s vast population of people, not only with schizophrenia but with other kinds of severe mental disorders too. Results of the LEAN intervention showed significant improvement in medication adherence, symptoms and functioning, reduction in the risk of relapses (defined as an overall and marked increase in symptoms assessed by the health professionals through interviewing patients and family members according to the 686 Program protocol) and re-hospitalizations among patients with schizophrenia [7]; however, the study did not collect sufficient cost data and failed to analyze and report the cost-effectiveness and cost-utility of the intervention.

As a decision to adopt a new strategy or service by health policy decision-makers depends on several factors such as the ability and willingness to pay for marginal health benefits, the effectiveness of the service, and the level of need in the community. Hence, there is a need for the development of a model that can be used to estimate the cost-effectiveness and cost-utility of this novel approach that could demonstrate the added value of the program over the existing program. Hence, the objective of this study is: (i) to develop an economic model to assess the cost and health outcomes of LEAN intervention from a societal perspective and validate the model and (ii) to assess the cost-effectiveness and cost-utility of the LEAN intervention versus the 686 Program alone.

## Methods/design

### Study design

This will be a cost-effectiveness analysis (CEA) and cost-utility analysis (CUA) of a randomized controlled trial that will compare people with schizophrenia who received the 686 Program alone (control group) with those who received the LEAN intervention along with the 686 program (intervention group) that delivered mobile phone text messaging (Table 1). CEA, the most commonly employed type of economic analysis [14] will compare the costs of averting the number of relapses and the number of re-hospitalizations in LEAN intervention as compared to the 686 program. The added value of the LEAN intervention over the 686 Program will be captured through the incremental cost-effectiveness ratio (ICER). For CUA, the effects of an intervention will be expressed as a measure of ‘utility’, commonly measured using the quality-adjusted life-year (QALY) [14]. The study will adopt a broad societal perspective on value, specifically to incorporate all health care costs such as direct medical cost of identifying individuals with schizophrenia and their medication adherence, and all health effects such as patient symptoms, functioning, relapses, and re-hospitalizations [15]. The indirect non-medical costs will include production losses related to reduced efficiency at work and absenteeism at work [16].

**Table 1:** Types of economic analysis and economic costs of the LEAN intervention

Type of analysis	Cost category	Costs	Outcome (effect)	Results expressed as:
CEA	• Cost of human resources • Cost of intervention • Cost of using medical resources	Monetary units (CNY)	Effect of intervention (number of relapses and re-hospitalizations averted	• Cost per relapse and re-hospitalization averted • ICER
CUA	• Cost of identifying individuals with schizophrenia and their medication adherence • Cost of assessing change in symptoms and functioning	Monetary units (CNY)	Healthy lifetime gained (QALYs)	Cost per QALY gained

*CEA* Cost-effectiveness analysis, *CUA* Cost-utility analysis, *CNY* Chinese yuan, *ICER* Incremental cost-effectiveness ratio, *QALY* Quality-adjusted life-year

The disease progression model as defined by McGorry et al. [17] that states different clinical staging of psychiatric disorders has been used to develop the basic design of the model. The final framework of the Markov model will be decided after the consultation with mental health experts, public health experts, and experts in economic modeling.

### Setting and duration

**Fig. 1:**
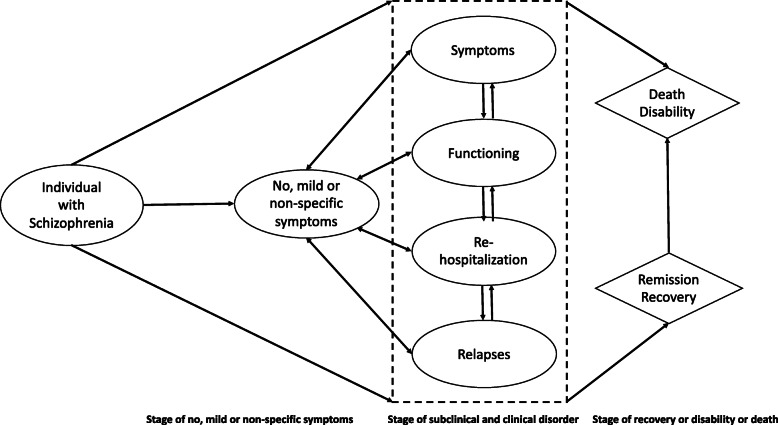
Basic design of Markov model illustrating different states of mental health through which an individual with schizophrenia could transition. Each state is associated with the cost and value of quality-adjusted life-year (QALY) gained. All states may also progress to death or disability

The LEAN trial was implemented in 9 rural townships (total population 356,900) of Liuyang municipality, Hunan Province, in central China. In this parent trial, the intervention group received LEAN intervention along with the 686 Program, while the control group received only the 686 Program that lasted from December 15, 2015 to June 15, 2016 for the duration of 6 months (Fig. 1).

### Study sample

Participants were randomly selected from the community-dwelling villagers having a primary diagnosis of schizophrenia in the parent study and were randomized 1:1 into two groups. Participants were selected if they were community-dwellers, were enrolled in the 686 Program, had a primary diagnosis of schizophrenia, were on oral psychotropic medications, and physically resided in 1 of 9 rural townships of Liuyang municipality. Participants were excluded from the study if they were hospitalized due to schizophrenia at the time of recruitment, had missed the most recent three consecutive past drug refills, or were physically incapable of using voice or text messaging. The primary outcome of LEAN trial was medication adherence assessed by unannounced home-based pill-counts; secondary outcomes included patient-level functioning assessed using 12-item World Health Organization Disability Adjustment Scale 2.0 (WHODAS 2.0) [18, 19], the severity of symptoms assessed by Clinical Global Impression in Schizophrenia (CGI-Sch) [20], relapse, and re-hospitalization. Outcome data were collected at baseline during the recruitment process and at 6 months after the implementation of LEAN. In addition to the outcome data collected as part of the trial, we will do additional fieldwork to collect program cost (i.e., cost data and effectiveness data) of the implemented LEAN intervention. Trained research assistants (RAs) will be responsible for conducting interview using survey questionnaire with the lay-health workers and family members to collect program cost data. RAs will also be responsible for obtaining the signed informed consent form from the participants. On the consent form, participants will be asked if they agree to use of their data and for permission for the research team to share relevant data with people from the university taking part in the research or from regulatory authorities, where relevant.

### Sample size

The required sample size of 258 participants was calculated in the parent study [7], which will also be used in the current economic evaluation. Given the nature of our planned cost-effectiveness study, a sample size of 258 participants will be sufficient and relevant for conducting interviews to assess costs thus meeting the purpose of our study.

### Data

#### Cost data

The original research collected little cost data. Additional fieldwork will be conducted to supplement the cost data that will include cost data on human resources (family members and lay health supporters, and health professionals – village doctors, mental health administrators, and psychiatrists), intervention (text message development, and maintenance of texting platform and texting), project management, medication cost incurred due to increased adherence, and use of medical resources (outpatient and inpatient care), and indirect non-medical costs (efficiency at work and absenteeism at work in the past 4 weeks), assessed with the second part of the Trimbos/iMTA questionnaire for Costs associated with Psychiatric Illness (TiC-P) [21]. Primary data will be collected from the study participants by an interview and clinical data will be extracted from the medical records (Table 2).

**Table 2:** Description of key model variables

Variables	Operational definition	Type of variable	Instrument used	Source of the data
*Demographic and epidemiologic variables*				
Population data	The initial number of people with a diagnosis of schizophrenia in each township	Continuous	Survey, records	Published literature, medical records, the 686 Program registry
Mortality rate	Incidence of death due to any reason	Continuous	Survey, records	Parent study, Medical record
Morbidity rate	Incidence of developing relapse	Continuous	Survey, records	Parent study, Medical record
Prevalence of Schizophrenia	National schizophrenia prevalence	Continuous	Survey, records	Published literature, China Health Statistical Yearbook
*Outcome variables*				
Medication adherence	The proportion of antipsychotic dosages taken over the past 30 days	Continuous	Unannounced pills count	Parent study, the 686 Program medication prescribing system
Relapse	Clinically significant exacerbation of psychotic symptoms	Dichotomous	Interview (self-report and/or caregiver report)	Parent study
Re-hospitalization	Re-hospitalization due to schizophrenia	Dichotomous	interRAI MH	Parent study, Medial record, field work
Functioning	Level of difficulty in accomplishing tasks and activities over the past 30 days	Ordinal	WHODAS 2.0	Parent study
Symptoms	Severity of psychotic symptoms	Ordinal	CGI-Sch	Parent study
*Program cost variables*				
Intervention cost	• Cost of training in preparation for LEAN • Cost of identifying individuals with schizophrenia • Cost of assessing medication adherence • Cost of message development and management • Cost of phones • Cost of texting to patients and lay-health supporters	Continuous	Cost estimating tool	Fieldwork to be conducted as part of the study, parent study
Human resource cost	Cost of family members, lay health supporters, and health professionals (village doctors, mental health administrators, and psychiatrists)	Continuous	Cost estimating tool	Fieldwork to be conducted as part of the study
Medication cost	Antipsychotics cost incurred due to increased adherence	Continuous	Cost estimating tool	Fieldwork to be conducted as part of the study
Indirect non-medical costs: • Efficiency at work Absenteeism at work	• Number of days with reduced efficiency due to feeling ill • Number of days absent from work	Continuous	TiC-P	Fieldwork to be conducted as part of the study

Parent study is the LEAN intervention

*interRAI MH* inter Resident Assessment Instrument-Mental Health, *WHODAS* World Health Organization Disability Assessment Schedule, *CGI-Sch* Clinical Global Impression in Schizophrenia, *TiC-P* Trimbos/iMTA questionnaire for Costs associated with Psychiatric Illness

Costs will be estimated using quantities of resource used and prices. The price will be estimated using existing market prices or shadow prices (the estimated price of a good or service for which no market price exists). Costs can be fixed (that cannot be changed) or variable (flexible and changes with productivity level); the sum of both these costs will be considered as the total cost. We will develop a standardized cost collection tool and will validate it before we collect new data. For the interpretation of costs of LEAN intervention, we will estimate the incremental change to total costs.

#### Effectiveness data

The effectiveness data will be extracted from published literature on risk-prediction. Published risk-prediction models will be used to estimate medication adherence [22] and the risk of developing relapses [23] and re-hospitalizations [24, 25].

### Outcomes

#### Effectiveness outcomes

The primary measure for cost-effectiveness will be the number of relapses and the number of re-hospitalizations avoided 6-month after the intervention. Relapses and re-hospitalizations cause a high burden to healthcare systems and patients and are used for different aims, such as a measure of cost control or quality of hospital care. There are no established criteria for defining relapse in schizophrenia. In our study, relapse will be defined as clinically significant exacerbation of psychotic symptoms evaluated by the investigators during interviews according to self-report or caregiver’s report [10]. The inter Resident Assessment Instrument-Mental Health (interRAI MH) assessment tool that has been validated in China will be used to assess the risk of re-hospitalization due to schizophrenia [26]. The interRAI MH includes a range of items measuring health service utilization, clinical and functional status, harm to self and others, social relationships, and vocational factors that assess the risk of re-hospitalizations [27, 28]. Study staff responsible for data collection will complete the assessment tool based on the interview, observation, and discussion with patients and family members. The study will also assess clinical/medical records to extract the data that will be valued in the model for predicting outcome measures. The secondary effectiveness measures will be medication adherence. The medication adherence will be measured using the proportion of dosage taken and good adherence will be defined as taking more than 80% of monthly pill counts, as stated in a previous study conducted among people with schizophrenia [29]. Adherence will be assessed using two unannounced home-based pill counts 30 days apart at the 6-month endpoint.

#### Utility outcomes

The measure for cost-utility will be Quality Adjusted Life Years (QALYs). QALY serves as a generic measure of health outcomes as it allows a combination of both morbidity, measuring the quality of life, and mortality, measuring the length of life, into a single score [30]. QALYs will be calculated using the 12-item version of the WHODAS 2.0 [18] that has been validated for the Chinese population [31, 32]. The WHODAS 2.0 measures daily functioning across six domains: cognitive (understanding and communicating), mobility (getting around), self-care, getting along with people, life activities and participation in society. Participants were asked to state the level of difficulty in accomplishing tasks and activities over the past 30 days, based on a 5-point ordinal scale (none, 1; mild, 2; moderate, 3; severe, 4; and extreme, 5) [33].

All data will be collected and managed using REDCap electronic data capture tool [34] hosted at the Sun Yat-sen University. To maintain confidentiality, all data collected as part of this study will be identified with the unique identification number allocated to the participants in LEAN trial.

### Model development and validation

#### Model development

**Fig. 2:**
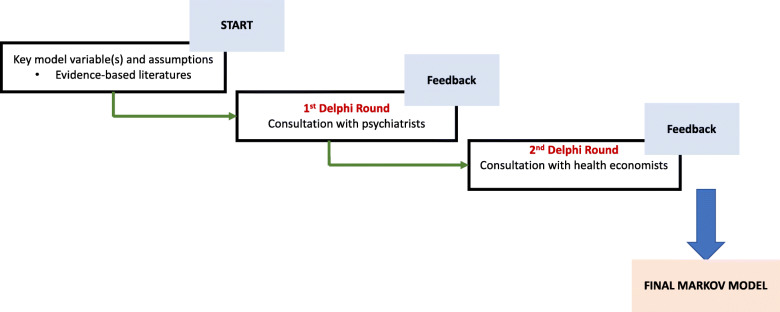
Delphi technique in model development

We will develop a Markov model [35] to compare outcome measures (e.g., medication adherence, symptoms, functioning, and QALYs) and costs of care in patients with schizophrenia approached with LEAN intervention plus 686 program versus 686 program alone. The model will employ Markov-type simulation of long-term health benefits, health care costs, and cost-effectiveness of the LEAN intervention. The iterative process of development of model structure and estimating model parameters will involve: reviewing of evidence-based literature, synthesizing data, consultation with health economists and statisticians, consultation with psychiatrists for expert opinion, and making some PAgs using the modified Delphi technique (Fig. 2) [36]. First-order Monte Carlo simulation [37] technique will be used to generate expected outcomes and costs of care for a hypothetical cohort of 10,000 patients.

#### Model validation

A framework developed by McCabe et al. to test the validity of the cost-effectiveness model will be used to validate the model in this study [38]. Validity will be assessed in terms of (i) the structure of the model (descriptive validity): ensuring that the possible pathways described by the model are feasible and sensible; (ii) the inputs of the model (internal validity): justification of the data utilized in the model; (iii) the results of the model (predictive validity): comparison of the modeled estimates with those produced in real life; and (iv) the value of the model to the decision maker: comparison of the structure, inputs and results of a model with those of existing cost-effectiveness models on treatment of mental health conditions [39, 40] and justification of differences.

### Data analysis

#### Incremental cost-effectiveness ratio (ICER)

A healthcare sector perspective of the cost will be estimated using ICER and their uncertainty. The ICER will be calculated as the cost incurred to gain one QALY:

$$ ICER=\left( CT- CC\right)/\left( ET- EC\right)=\Delta C/\Delta E $$

where, CT and ET are the cost and effect of a new health program i.e., LEAN intervention in this study and CC and EC are the cost and effect of the baseline comparator i.e., the community-based mental health program. Distribution of incremental costs and effects will be presented graphically on a cost-effectiveness plane, to show the estimated joint distribution of incremental costs against incremental effects. To avoid inconsistencies in the conclusions and account for the timing of events, we will apply a discounted rate of 3%; this is the rate typically used in the cost-effectiveness analysis [41].

#### Analysis of uncertainty

One-way sensitivity analyses will be undertaken to determine which parameters have the most impact on the results. To analyze the overall uncertainty in the model, we will employ a Probabilistic Sensitivity Analysis (PSA) in which every parameter of the model will be assigned a statistical distribution. With this technique, we will obtain the distributions of costs, health outcome values, and the resulting cost-effectiveness ratios, that will include estimation of 95% uncertainty intervals. Key input parameters to be examined will include, but will not be limited to, discount rate, intervention costs, non-adherent rates, and unit costs of schizophrenia-related morbidities. Results from the analysis will be presented in cost-effectiveness scatter plots. TreeAge Pro 2018 Software will be used as an analytical tool [42].

## Discussion

The purpose of this study will be to assess the cost-effectiveness and cost-utility of the LEAN intervention as compared to the nationwide community-based mental health program. With this study, we aim to investigate the cost-effectiveness and cost-utility of LEAN intervention, see an improvement in the patient’s quality of life, achieve a reduction in the relapse of cases and re-hospitalization number, achieve an increase in the medication adherence, and be able to report a decrease in the health care costs. This study will provide a novel economic model to evaluate the cost-effectiveness and cost-utility of alternative public health interventions for schizophrenia and possibly other mental disorders. Further, the model can be used in the development of mental health programs and measuring cost-effectiveness of those program in different health care settings within China and in other resource-limited countries with mental health workforce gap.

If the cost-effectiveness and cost-utility of the LEAN intervention is demonstrated, professionals might use it in the primary health care settings of China with the expectation of improving quality of life of population with schizophrenia, decreasing incidence of disease comorbidities, and reducing social costs to the patient and his/her family. Thus, the findings from our study will be of interest to clinicians and others interested in costs and outcomes of mental health services among patients with schizophrenia or other severe mental disorders.

The strength of our study is that the LEAN intervention has been designed as a pragmatic effectiveness trial. In this type of design, the interventions reflect what may happen in practice and the results are often more generalizable and hence preferable for economic evaluation [43]. The economic model developed as part of this study will be flexible with a standardized framework, permitting the economic evaluation of schizophrenia management strategies in different health care settings of China. Our study will also have some limitations. Although the study by Xu et al. reported superior efficacy of LEAN in increasing medication adherence and substantial reduction in the risk of relapse and re-hospitalization [7], there is no any randomized controlled trial conducted for the nationwide program yet. Hence, the efficacy of nationwide program in unknown, making our comparison perhaps debatable. Further, due to the generalizability constraint of the Chinese context, the model developed in this study might need some adaptation before it could be used to other resource-limited settings with similar context. Despite these limitations, we believe our study has important clinical and economic implications.

## Data Availability

Data will be available from the corresponding author on reasonable request.

## Abbreviations

CEA: Cost-effectiveness analysis
CUA: Cost-utility analysis
CGI-Sch: Clinical Global Impression in Schizophrenia
HBM: Health Belief Model
ICER: Incremental cost-effectiveness ratio
interRAI-MH: Inter Resident Assessment Instrument-Mental Health
LEAN: Lay health supporters, E-platform, Award, and iNtegration
PSA: Probabilistic sensitivity analysis
TiC-P: Trimbos/iMTA questionnaire for Costs associated with Psychiatric Illness
QALY: Quality Adjusted Life Year
WHODAS: World Health Organization Disability Assessment Schedule

## References

[1] Zhang F, Zhao J. China is Prepared to Fight Against Emerging Mental Health Disorders? Int J Emerg Mental Health Human Resilience. 2015;17:628–34 ISSN 1522-4821.

[2] Ran M-S, Weng X, Liu Y-J, Zhang T-M, Thornicroft G, Davidson L (2017). Severe mental disorders in rural China: a longitudinal survey. Lancet.

[3] Charlson FJ, Baxter AJ, Cheng HG, Shidhaye R, Whiteford HA (2016). The burden of mental, neurological, and substance use disorders in China and India: a systematic analysis of community representative epidemiological studies. Lancet.

[4] Hu X, Rohrbaugh R, Deng Q, He Q, Munger KF, Liu Z (2017). Expanding the mental health workforce in China: narrowing the mental health service gap. Psychiatr Serv.

[5] Wang X, Zhang W, Ma N, Guan L, Law SF, Yu X (2016). Adherence to antipsychotic medication by community-based patients with schizophrenia in China: a cross-sectional study. Psychiatr Serv.

[6] Paganini S, Teigelkötter W, Baumeister H (2016). Economic evaluations of internet-and mobile-based interventions for depression: a systematic review. Euro Health Psychol.

[7] Xu DR, Xiao S, He H, Caine ED, Gloyd S, Simoni J (2019). Lay health supporters aided by mobile text messaging to improve adherence, symptoms, and functioning among people with schizophrenia in a resource-poor community in rural China (LEAN): a randomized controlled trial. PLoS Med.

[8] Xu DR, Gong W, Caine ED, Xiao S, Hughes JP, Ng M (2016). Lay health supporters aided by a mobile phone messaging system to improve care of villagers with schizophrenia in Liuyang, China: protocol for a randomised control trial. BMJ Open.

[9] Becker MH (1974). The health belief model and personal health behavior. Health Educa Monogr.

[10] Xiao J, Mi W, Li L, Shi Y, Zhang H (2015). High relapse rate and poor medication adherence in the Chinese population with schizophrenia: results from an observational survey in the People’s republic of China. Neuropsychiatr Dis Treat.

[11] Zhou Y, Ning Y, Rosenheck R, Sun B, Zhang J, Ou Y (2016). Effect of living with patients on caregiver burden of individual with schizophrenia in China. Psychiatry Res.

[12] Good BJ, Good M-JD (2012). Significance of the 686 program for China and for global mental health. Shanghai Arch Psychiatry.

[13] Liang D, Mays VM, Hwang W-C (2017). Integrated mental health services in China: challenges and planning for the future. Health Policy Plan.

[14] Smith PG, Morrow RH, Ross DA (2015). Intervention costing and economic analysis. Field Trials of Health Interventions: A Toolbox 3rd edition.

[15] Jönsson B. Ten arguments for a societal perspective in the economic evaluation of medical innovations. Eur J Health Econ. 2009;10(4):357–9. 10.1007/s10198-009-0173-2. 10.1007/s10198-009-0173-2 19618224

[16] Smit HFE, Cuijpers P, Oostenbrink J, Batelaan NM, de Graaf R, Beekman AJ (2006). Costs of nine common mental disorders: implications for curative and preventive psychiatry. J Ment Health Policy Econ.

[17] McGorry PD, Hickie IB, Yung AR, Pantelis C, Jackson HJ (2006). Clinical staging of psychiatric disorders: a heuristic framework for choosing earlier, safer and more effective interventions. Aust N Z J Psychiatry.

[18] Üstün TB, Kostanjsek N, Chatterji S, Rehm J (2010). Measuring health and disability: Manual for WHO disability assessment schedule WHODAS 2.0: World Health Organization.

[19] Üstün TB, Chatterji S, Kostanjsek N, Rehm J, Kennedy C, Epping-Jordan J (2010). Developing the World Health Organization disability assessment schedule 2.0. Bull World Health Org.

[20] Haro JM, Kamath SA, Ochoa SO, Novick D, Rele K, Fargas A (2003). The clinical global impression–schizophrenia scale: a simple instrument to measure the diversity of symptoms present in schizophrenia. Acta Psychiatr Scand.

[21] Hakkaart-van Roijen L, Van Straten A, Donker M, Tiemens B (2002). Manual Trimbos/iMTA questionnaire for costs associated with psychiatric illness (TiC-P).

[22] Byerly MJ, Nakonezny PA, Rush AJ (2008). The brief adherence rating scale (BARS) validated against electronic monitoring in assessing the antipsychotic medication adherence of outpatients with schizophrenia and schizoaffective disorder. Schizoph Res.

[23] Vigod SN, Kurdyak PA, Seitz D, Herrmann N, Fung K, Lin E (2015). READMIT: a clinical risk index to predict 30-day readmission after discharge from acute psychiatric units. J Psychiatr Res.

[24] Hasan O, Meltzer DO, Shaykevich SA, Bell CM, Kaboli PJ, Auerbach AD (2010). Hospital readmission in general medicine patients: a prediction model. J Gen Intern Med.

[25] van Walraven C, Dhalla IA, Bell C, Etchells E, Stiell IG, Zarnke K (2010). Derivation and validation of an index to predict early death or unplanned readmission after discharge from hospital to the community. CMAJ..

[26] Chan CLF, Lai CKY, Chi I (2014). Initial validation of the Chinese interRAI mental health in people with psychiatric illness. Int J Psychiatry Clin Pract.

[27] Hirdes JP, Marhaba M, Smith TF, Clyburn L, Mitchell L, Lemick RA (2000). Development of the resident assessment instrument–mental health (RAI-MH). Hosp Q.

[28] Perlman CM, Hirdes JP, Vigod S. Psychiatric rehospitalization: development of a person-level indicator for care planning and quality assurance. Prim Care Companion CNS Disord. 2015;17(4):10.4088/PCC.15m01784. 10.4088/PCC.15m01784. 10.4088/PCC.15m01784 PMC4664575 26693047

[29] Kim J, Ozzoude M, Nakajima S, Shah P, Caravaggio F, Iwata Y (2019). Insight and medication adherence in schizophrenia: an analysis of the CATIE data. Neuropharmacology..

[30] Prieto L, Sacristán JA (2003). Problems and solutions in calculating quality-adjusted life years (QALYs). Health Qual Life Outcomes.

[31] Cheung MKT, Hung ATF, Poon PKK, Fong DYT, Li LSW, Chow ESL (2015). Validation of the World Health Organization assessment schedule II Chinese traditional version (WHODAS II CT) in persons with disabilities and chronic illnesses for Chinese population. Disabil Rehabil.

[32] Chiu T-Y, Yen C-F, Chou C-H, Lin J-D, Hwang A-W, Liao H-F (2014). Development of traditional Chinese version of World Health Organization disability assessment schedule 2.0 36–item (WHODAS 2.0) in Taiwan: validity and reliability analyses. Res Dev Disabil.

[33] WHO Disability Assessment Schedule 2.0 (WHODAS 2.0). https://www.who.int/classifications/icf/whodasii/en/. Accessed 22 July 2019.

[34] Harris PA, Taylor R, Thielke R, Payne J, Gonzalez N, Conde JG (2009). Research electronic data capture (REDCap)—a metadata-driven methodology and workflow process for providing translational research informatics support. J Biomed Inform.

[35] Sonnenberg FA, Beck JR (1993). Markov models in medical decision making: a practical guide. Med Decis Mak.

[36] Park RE, Fink A, Brook RH, Chassin MR, Kahn KL, Merrick NJ (1986). Physician ratings of appropriate indications for six medical and surgical procedures. Am J Public Health.

[37] Halpern EF, Weinstein MC, Hunink MGM, Gazelle GS (2000). Representing both first-and second-order uncertainties by Monte Carlo simulation for groups of patients. Med Decis Mak.

[38] McCabe C, Dixon S (2000). Testing the validity of cost-effectiveness models. Pharmacoeconomics..

[39] Palmer CS, Brunner E, Ruíz-Flores LG, Paez-Agraz F, Revicki DA (2002). A cost-effectiveness clinical decision analysis model for treatment of schizophrenia. Arc Med Res.

[40] Edwards NC, Rupnow MFT, Pashos CL, Botteman MF, Diamond RJ (2005). Cost-effectiveness model of long-acting risperidone in schizophrenia in the US. Pharmacoeconomics..

[41] Drummond MF, Sculpher MJ, Claxton K, Stoddart GL, Torrance GW (2015). Methods for the economic evaluation of health care programmes: Oxford university press.

[42] TreeAge (2018). TreeAge Pro 2018.

[43] Ramsey S, Willke R, Briggs A, Brown R, Buxton M, Chawla A (2005). Good research practices for cost-effectiveness analysis alongside clinical trials: the ISPOR RCT-CEA task force report. Value Health.

